# Effects of the Symbiotic *Chlorella variabilis* on the Host Ciliate *Paramecium bursaria* Phenotypes

**DOI:** 10.3390/microorganisms12122537

**Published:** 2024-12-09

**Authors:** Yuuki Kodama, Masahiro Fujishima

**Affiliations:** 1 Institute of Agricultural and Life Sciences, Academic Assembly, Shimane University, Nishikawatsu-cho 1060, Matsue-shi 690-8504, Shimane, Japan; 2 Research Center for Thermotolerant Microbial Resources, Yamaguchi University, Yoshida 1677-1, Yamaguchi 753-8512, Yamaguchi, Japan; fujishim@yamaguchi-u.ac.jp

**Keywords:** *Chlorella* sp., *Chlorella variabilis*, cytoplasmic crystal, digestive vacuole, endosymbiosis, heat tolerance, mitochondria, *Paramecium bursaria*, perialgal vacuole, photoaccumulation, trichocyst

## Abstract

*Paramecium bursaria*, a ciliated protist, forms a symbiotic relationship with the green alga *Chlorella variabilis*. This endosymbiotic association is a model system for studying the establishment of secondary symbiosis and interactions between the symbiont and its host organisms. Symbiotic algae reside in specialized compartments called perialgal vacuoles (PVs) within the host cytoplasm, which protect them from digestion by host lysosomal fusion. The relationship between *P. bursaria* and symbiotic *Chlorella* spp. is characterized by mutualism, in which both organisms benefit from this association. Furthermore, symbiotic algae also influence their host phenotypes, and algae-free *P. bursaria* can be obtained through various methods and reassociated with symbiotic algae, making it a valuable tool for studying secondary endosymbiosis. Recent advancements in genomic and transcriptomic studies on both hosts and symbionts have further enhanced the utility of this model system. This review summarizes the infection process of the symbiotic alga *C. variabilis* and its effects on the algal infection on number of host trichocysts, mitochondria, cytoplasmic crystals, total protein amount, stress responses, photoaccumulation, and circadian rhythms of the host *P. bursaria*.

## 1. Introduction

Endosymbiosis theory refers to the concept that certain organelles within eukaryotic cells, such as mitochondria and plastids, originate from free-living prokaryotic organisms that establish a permanent residence within another cell, leading to a mutually beneficial relationship [1]. Symbiotic relationships are common in nature and can be mutualistic or parasitic. The term “symbiosis” is frequently employed to describe mutually beneficial interactions; however, it encompasses any relationship between two organisms in which they coexist, regardless of whether the association is beneficial, neutral, or detrimental [2]. In marine ecosystems, invertebrates frequently form symbiotic partnerships with photosynthetic dinoflagellate algae of the genus *Symbiodinium*. These mutualistic associations are common among *Cnidarians*, including various corals, sea anemones, and jellyfish [3]. In contrast, freshwater environments host endosymbiotic relationships involving *Chlorella* spp. or similar algae. These algal symbionts are found in numerous protists, such as *Paramecium bursaria*, *Euplotes daidaleos*, *Difflugia* sp., *Climacostomum virens*, *Coleps hirtus*, *Stentor polymorphus*, *Ophrydium* sp., *Frontonia* sp., *Mayorella viridis* [4,5], *P. chlorelligerum* [6], and *Tetrahymena utriculariae* [7,8]. Understanding endosymbiosis facilitates the elucidation of eukaryotic organelle origins, such as chloroplasts and mitochondria, as well as the elucidation of interactions between organisms in endosymbiotic relationships [9].

**Figure 1:**
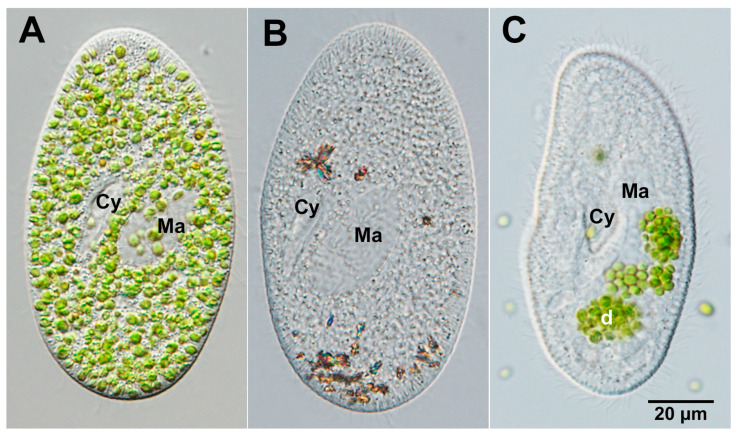
Differential interference contrast (DIC) micrographs of *P. bursaria*, a model organism for investigating the induction of secondary symbiosis. (**A**) *P. bursaria* harboring several hundred symbiotic *C. variabilis*. (**B**) Artificial algae-removed *P. bursaria* from (**A**). (**C**) Shows (**B**) fixed 30 s after mixing with the symbiotic *C. variabilis* isolated from (**A**). The upper and lower sides of the photographs are the anterior and posterior sides, respectively. Cy, cytopharynx; Ma, macronucleus; d, digestive vacuole (DV)-containing algae. Kodama, unpublished micrographs.

The protist *Paramecium bursaria* is a member of the diverse phylum Ciliophora, and its most famous feature harbors several hundred endosymbiotic algae in its cytoplasm (Figure 1A). Recent studies using molecular techniques have shown that most *P. bursaria* specimens contain symbiotic algae from either American or European groups [10]. These symbionts have been identified as *Chlorella variabilis* and *Micractinium reisseri*, both of which belong to the Chlorellaceae family within the class Trebouxiophyceae [10]. The perialgal vacuole (PV) membrane in *P. bursaria* is a specialized compartment that encloses symbiotic algae. This compartment is derived from the host digestive vacuole (DV) membrane and is known as the symbiosome membrane, which separates the endosymbiont from the host cell cytoplasm and facilitates metabolic exchange and protection of the endosymbiont from the host defense mechanisms [11,12,13] and infection of chlorovirus [14]. Symbiotic algae of *P. bursaria* are transferred to host daughter cells during host cell division and maintained during conjugation [15]. Interestingly, the population size of symbiotic algae within the host cell remains several hundred cells in the presence of light and nutrients [16]. *P. bursaria* regulates the abundance of its symbionts through glutamine supply [9].

The relationship between *P. bursaria* and symbiotic *Chlorella* spp. is characterized by mutualism, in which both organisms benefit from this association [17,18,19,20,21]. For example, the host *P. bursaria* provides a protective habitat for *Chlorella* spp., whereas algae contribute to ciliate nutrition through photosynthesis [22]. The presence of symbiotic *Chlorella* spp. influences the circadian rhythms and stress responses of *P. bursaria*, including protection against UV radiation, high temperatures, chemicals, and oxidative stress [23,24,25]. Although this mutually beneficial relationship between *P. bursaria* and its symbiotic algae is classified as facultative mutualism, both algae-free *P. bursaria* cells (also known as algae-removed cells; Figure 1B) and isolated symbiotic algae can grow independently. The production of algae-free *P. bursaria* from algae-bearing cells is a straightforward process. This can be achieved through rapid fission [26], cultivation in darkness [15,27,28], X-ray irradiation [29], or treatment with various photosynthetic inhibitors, such as paraquat [30], 3-(3,4-dichlorophenyl)-1,1-dimethylurea (DCMU) [31], or a protein synthesis inhibitor cycloheximide in eukaryotes [11,32]. By cultivation in darkness or treatment with cycloheximide, algal digestion by the host’s lysosomal fusion to the PV membrane is induced [11]. Once obtained, algae-free *P. bursaria* can be cultivated under feeding conditions even without symbiotic algae. In addition, algae-free *P. bursaria* can also be reassociated into native and free-living *Chlorella* spp. by mixing them together, as shown in Figure 1C. These notable characteristics exhibited by *P. bursaria* have been well-documented for an extended period [33,34].

Recently, the nuclear genome of the symbiotic *C*. *variabilis* [35] and the macronuclear genome of the host *P*. *bursaria* were identified [36]. In addition, the amino acid and codon usage of *P. bursaria* was investigated using transcriptome data [37]. RNA interference (RNAi) knockdown of selected genes can be effectively accomplished by administering *Escherichia coli* expressing double-stranded RNA complementary to the *Paramecium* DNA sequence to paramecia [38,39,40]. This methodology has been employed for RNAi induction in *P. bursaria* [9,41]. In a previous study, transcriptome analysis (RNA-seq) of *P. bursaria* in the presence or absence of symbionts was performed to investigate the genes associated with endosymbiosis. Gene expressions between algae-containing and algae-free *P. bursaria* were compared to determine the genetic mechanisms underlying endosymbiosis establishment. In the case of algae-containing *P. bursaria*, several genes exhibited reduced expression. These included genes encoding glutathione S-transferase, trans-2-enoyl-CoA, ribosomal proteins, and aminotransferases, all of which were found among the differentially expressed genes. Conversely, genes encoding transcriptional activator Myb-related proteins, Hsp70, and signal transduction histidine kinase showed increased expression. This study provides a comprehensive sequence resource for future investigations of *P. bursaria* [42]. Consequently, *P. bursaria* is emerging as a model organism for investigating the induction of secondary symbiosis, which refers to the process by which a eukaryotic host cell acquires another eukaryotic cell that has previously undergone primary symbiosis as an endosymbiont [43].

## 2. Methods

### 2.1. Study Design

This systematic review was conducted on published articles reporting the endosymbiotic relationships between *P. bursaria* and *Chlorella* spp. using light and electron microscopy and molecular techniques.

### 2.2. Search Strategy

PubMed and Google Scholar were searched for articles and reviews published in English, German, or Japanese up to 2024. The search keywords were “*P. bursaria*” and “*Paramecium bursaria*”. Furthermore, a systematic review of 1013 abstracts from articles and reviews published between 2020 and 2024 was conducted, identified using the keywords “endosymbiosis”, “endosymbiont”, “symbiosis”, “symbiont”, “infection”, “kleptoplasty”, and “karyoklepty”. A list of articles and reviews was prepared using the Web of Science database. Full-text articles were downloaded from Shimane University Library Resources.

## 3. Re-Endosymbiosis Process of Symbiotic *Chlorella variabilis* to the Algae-Free *P. bursaria*

Artificial re-endosymbiosis experiments of algae-free *P. bursaria* cells and *Chlorella* species were already performed 65 years ago by Siegel and Karakashian [34]. When algae-free *P. bursaria* cells were mixed with symbiotic algae isolated from algae-bearing *P. bursaria* cells, most of the algae ingested by the host were digested, but some escaped and survived in the host cytoplasm [18]. However, the specifics of this re-endosymbiotic process remained unclear. Therefore, elucidation of the algal re-endosymbiosis process in algae-free *P. bursaria* cells via host phagocytosis was attempted. To examine the algal re-endosymbiosis process, the fate of ingested algae in the host DVs was monitored every 10 s. Algae-free *P. bursaria* cells were mixed with symbiotic algae isolated from algae-bearing *P. bursaria* cells for 1.5 min, followed by washing and fixing the mixture. The cells were subsequently analyzed by DIC and transmission electron microscopy (TEM). In some observations, Gomori’s staining method [44] was used to detect *Paramecium* lysosome-specific acid phosphatase (AcPase) activity. AcPase is an enzyme marker of lysosomal activity [45]. Gomori’s staining method has traditionally been used for the localization of AcPase activity, but it is still used today for the histochemistry of plants, animals, and protists [13,46,47]. As a result, the following four key cytological phenomena were identified as necessary for establishing endosymbiosis [48,49,50,51].

**Figure 2:**
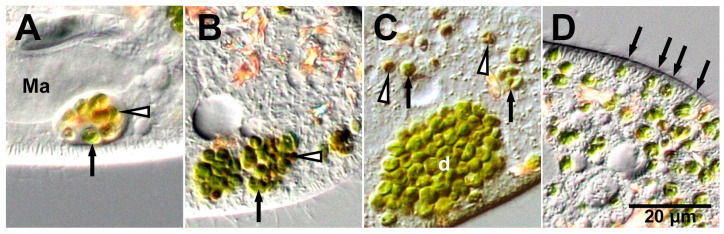
DIC micrographs of the re-endosymbiosis process of symbiotic *C. variabilis* to algae-free *P. bursaria*. (**A**) DV-IIIb was observed 19 min after mixing algae-free *P. bursaria* and symbiotic *C. variabilis* isolated from algae-bearing cells. Digested faint yellow algae (white arrowhead) and undigested green algae (arrow) coexisted in the same DV. (**B**) Several DV-IVbs were detected within 30 min of mixing with algae-free *P. bursaria* and the isolated symbiotic *Chlorella* sp. Digested brownish algae (white arrowhead) and undigested green algae (arrow) coexisted in the same DV. (**C**) Large DV (d) containing a large number of algae observed 1 h after mixing with algae-free *P. bursaria* and isolated symbiotic *Chlorella* sp. Green (arrow) and brownish (white arrowhead) algae budded from the DV one cell at a time. (**D**) Single green algae adhering below the host *Paramecium* cortex (arrow) observed 3 h after mixing with algae-free *P. bursaria* and the isolated symbiotic *Chlorella* sp. Ma, macronucleus; d, DV. Kodama, unpublished micrographs.

The first phenomenon is the temporary resistance of algae within the DV, where both the host acidosomes and lysosomes are fused. During algal re-endosymbiosis, four stages of DVs, namely, DV-I, DV-II, DV-III, and DV-IV, were observed, and the DV stages were classified based on their morphology and pH inside the DVs [52]. Upon mixing with algae-free *P. bursaria* cells, one or more algal cells moved through the *P. bursaria* cytopharynx and were endocytosed inside the initial DV, DV-I. DV-I exhibited a distinctly visible DV membrane and contained exclusively green algae. The pH of DV-I is neutral. Condensed and acidified DV-II appeared 30 s − 1 min after algal mixing, and the DV membrane was barely visible. Although the pH inside DV-II drops to 2.4–3, the algae did not change its morphology and retained its green color after acidosome–DV fusion. Even when the symbiotic *Chlorella* sp. isolated from algae-bearing *P. bursaria* was subjected to acidic buffer treatment for 1 h, it retained its infectivity to algae-free *P. bursaria* (Kodama and Fujishima, unpublished data). This suggests that algae are likely resistant to acidified DVs. Host lysosomal fusion to DVs occurs in approximately 2–3 min, accompanied by an increase in intra-DV pH and the formation of an enlarged DV-III. Some algae in DV-III begin to be digested, and as a result, the color of the algae in the DV is either digested faint yellow or undigested green. Thus, whether the algae in the DVs have been digested depends on the strain of *Chlorella* sp. but can be determined by the change in color of the algae. The DV-III stage was further subdivided into three substages: DV-IIIa, which exclusively contained undigested green algae; DV-IIIb, which contained both digested faint yellow and undigested green algae (Figure 2A); and DV-IIIc, which contained only digested faint yellow algae. In the final stage, DV-IV, the DV size was reduced to the same extent as in DV-II, rendering the DV membrane barely visible under a DIC microscope. The digestion of some algae was further advanced during differentiation into DV-IV from DV-III; therefore, digested brown and undigested green algae were observed in DV-IV. This stage was observed 20–30 min after algal mixing. DV-IV was also subdivided into three substages: DV-IVa, which exclusively contained undigested green algae; DV-IVb, which contained both undigested green and digested brownish algae (Figure 2B); and DV-IVc, which exclusively contained digested brownish algae. Some algae in DV-IIIb and DV-IVb remained undigested and retained their green coloration even when digested algae coexisted inside the same DVs. It was then clarified that the digestive enzyme resistance of the algae was contingent on the photosynthetic activity of the isolated *Chlorella* sp. prior to their integration with algae-free *P. bursaria* [53]. When symbiotic algae isolated from algae-bearing paramecia were maintained in constant darkness for 24 h before introduction into algae-free *P. bursaria*, the majority of algae underwent digestion in the host DVs, thereby impeding the re-establishment of endosymbiosis [53].

The second phenomenon is the appearance of algae from DV into the cytoplasm of the host. Thirty minutes after the combination of algae-free *P. bursaria* and the isolated symbiotic *Chlorella* sp., the algae began budding from DV-IVb because of membrane budding into the cytoplasm of *P. bursaria*. Both undigested and digested algae have the ability to bud from DVs (Figure 2C) [51]. Additionally, polystyrene latex spheres and yeast *Saccharomyces cerevisiae* with diameters ≥3 μm are capable of budding from DVs by one sphere or one cell each [54]. Recently, Obayashi and Kodama succeeded in capturing a video of the moment yeast buds from the DV membrane one cell at a time [55]. However, this budding is not observed when bacteria, India ink, or 0.81 μm-diameter polystyrene latex spheres are ingested by DVs [51]. These results suggest that *P. bursaria* can recognize the size of its contents within a DV via an unknown mechanism. The dynamin inhibitor Dynasore significantly inhibits DV budding, indicating that dynamin plays a role in this process [54]. To elucidate the budding process, the isolated symbiotic algae were mixed with equal volumes of fluorescent 0.20 μm microspheres during algal re-endosymbiosis. Fluorescence of the microspheres was not observed near the PV membrane, as anticipated, but was detected in budding DVs containing both undigested green and digested algae. Moreover, the percentage of algal re-endosymbiosis was significantly diminished in the presence of 0.20 μm microspheres. These observations demonstrate that the host *P. bursaria* achieved the budding of algae individually from the membranes of DVs without microspheres. Moreover, this experiment utilizing small-diameter microspheres indicated that intimate contact between the DV membrane and algal cell wall was necessary to establish algal endosymbiosis [56].

The third phenomenon is that the DV membrane undergoes a transformation into the PV membrane. Subsequent to the budding of DV-IV membrane containing algae, DV membranes encompassing an undigested single green alga differentiate into PV membranes, thereby conferring protection against host lysosomal fusion [51,57,58]. To elucidate the temporal sequence of PV membrane differentiation from the DV membrane, algae-free *P. bursaria* cells were mixed with isolated symbiotic algae, washed out uningested algae completely, chased, and, thereafter, fixed at various times of observation. Subsequently, lysosomal AcPase activity in the DVs or PVs enclosing algae (alga) was examined by Gomori’s staining. Neither DV-I nor DV-II exhibited AcPase activity, whereas it was observed in the 3-min-old DV-III. All the DV-IV enclosing algae exhibited AcPase activity 0.5 h after DV formation. Algal escape from the DVs by budding of the DV membrane was initiated at 0.5 h, as previously described. In the budding DV membrane, each alga was enclosed by a thin black layer, indicating a positive AcPase activity within the membrane. Among the DVs, those containing a single green alga moved quickly and attached to the host *Paramecium* cortex. The vacuoles attached below the host cell cortex displayed no AcPase activity. The emergence of the initial budding alga from DV-IV and its subsequent attachment below the host *Paramecium* cortex occurred 30 and 45 min after mixing, respectively. These observations indicate that differentiation of the PV membrane from the DV-IV membrane occurs within 15 min of algal budding from the host DV because it is known that the PV membrane does not show AcPase activity [13,58]. Subsequently, monoclonal antibodies (mAbs), which are specific to the DV membrane of *P. bursaria,* were developed. The mAbs do not interact with the PV membrane, suggesting that the membranes exhibit substantial differences (Kodama and Fujishima, unpublished data). Limited information is available regarding the composition of the PV membrane; however, the localization of oligosaccharides in close proximity to the symbiotic *C. variabilis* in *P. bursaria* has been previously demonstrated [59]. Symbiotic algal samples enclosed in a PV membrane were found to contain oligosaccharides, whereas these compounds were not present in isolated symbiotic algae or algae enclosed in DV membranes. These observations offer a significant new understanding of the differences in the chemical components of DV and PV membranes [59].

The fourth phenomenon is the undigested (i.e., successful symbiosis) algae enclosed in the PV membrane positioned below the *Paramecium* cortex, where mitochondria and trichocysts are present (Figure 2D) [12,51,60,61]. This phenomenon is discussed in more detail in Section 5.2.

## 4. Microorganisms Capable of Symbiosis with Algae-Free *P. bursaria* Cells

In a previous study, free-living algal strains *Chlorella sorokiniana* strain NIES-2169 and *Parachlorella kessleri* strain C-531 were maintained in algae-free *P. bursaria* for more than two years after algal introduction [62]. In addition, *Chlorella variabilis* strain NC64A, isolated from algae-bearing *P. bursaria* approximately 60 years ago and cultivated outside the host for a long time [63], also demonstrated infectivity and was continuously maintained [64]. Although the lasting establishment of stable endosymbiosis appears to be restricted to *Chlorella* spp., various potential symbionts or parasites such as bacteria (*Pseudomonas* sp.), yeast (that is *Yarrowia lipolytica* and *Rhodotorula rubra*), and other green algae (that is *Scenedesmus* sp.), are able to infect *P. bursaria* [65,66,67]. Cyanobacteria (*Synechocystis* sp. strain PCC 6803) introduced into algae-free cells of *P. bursaria* were maintained even after continuous propagation of the ciliate for over 1 year [68]. However, the detailed infection process of these organisms in algae-free *P. bursaria* remains unknown, and some are considered to be contaminants [66].

**Figure 3:**
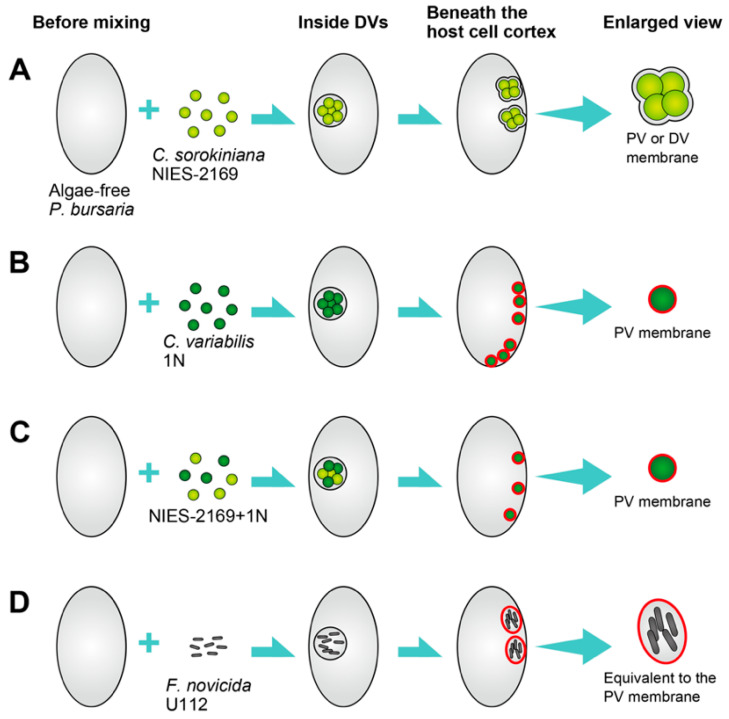
This diagram shows the fate of algae-free *P. bursaria* after ingestion of (**A**) free-living *Chlorella sorokiniana* strain NIES-2169, (**B**) native *Chlorella variabilis* strain 1N isolated from algae-bearing *P. bursaria*, (**C**) a mixture of NIES-2169 and 1N, and (**D**) pathogenic bacteria *F. novicida* strain U112. The blue line represents the PV membrane. The figure was taken from [69] with permission. The color of the PV membrane has changed from blue in the original figure to red.

Kodama and Endoh [69] examined the infection processes of the free-living *Chlorella* species *C. sorokiniana* and the native symbiotic *C. variabilis* in algae-free *P. bursaria* by observing the outcomes of these ingested algae. This was accomplished by exposing algae-free *P. bursaria* to three distinct algal inoculum types: *C. sorokiniana* alone, *C. variabilis* alone, or a combination of *C. sorokiniana* and *C. variabilis* [69]. The infection process for the various strains is shown in Figure 3. This depicts the free-living *C. sorokiniana* strain NIES-2169 (Figure 3A), native symbiotic *C. variabilis* strain 1N (Figure 3B), both *C. sorokiniana* and *C. variabilis* (Figure 3C), and *Francisella novicida* strain U112 (Figure 3D) after being taken up by the algae-free *P. bursaria*. The diagram representing U112 infection was based on the findings in [70]. *C. sorokiniana* cells are ingested via the host cytopharynx and subsequently enveloped by a DV membrane. The initial DV membrane was subjected to host cytoplasmic streaming, during which some algae were digested. Some *C. sorokiniana* cells were able to escape host digestion and localize below the host *Paramecium* cortex by wrapping the DV membrane, which encloses multiple cells (Figure 3A). This membrane has the characteristics of both the DV and PV membranes. As a characteristic of a DV membrane, it contains multiple algae and flows through the host cytoplasmic streaming [69], whereas as a characteristic of a PV membrane, it adheres to the host *Paramecium* cortex [57]. Additionally, host mitochondria are in close proximity to the membrane surrounding *C. sorokiniana*, which is consistent with the characteristics of the PV membrane [69]. The *C. sorokiniana* was maintained in the *P. bursaria* cell for several weeks [69]. The native symbiotic *C. variabilis* cells were enclosed within the DV membrane (Figure 3B). Similar to *C. sorokiniana*, the initial DV membrane encompassing multiple *C. variabilis* cells was exposed to host cytoplasmic streaming, leading to the digestion of some algae during this process. Some *C. variabilis* cells evaded host digestion and were observed below the *Paramecium* cortex. In contrast, in *C. variabilis*, each alga was individually situated below the host *Paramecium* cortex, suggesting encapsulation within the PV membranes, as depicted by the blue membrane in Figure 3B. When *C. sorokiniana* and *C. variabilis* were introduced together, they were both present within the same DV membrane (Figure 3C). Although some cells from both strains avoided host digestion, only *C. variabilis* cells encased in PV membranes successfully infected *P. bursaria* and established symbiotic relationships [69]. *Francisella novicida* is a Gram-negative, facultative intracellular pathogen closely related to *F. tularensis*, the causative agent of tularemia [71]. *F. novicida* strain U112 has also been found to be ingested and enclosed within the DV membrane. The U112 cells demonstrated the capacity to infect *P. bursaria* and were localized below the host cortex in a manner similar to that observed in *C. sorokiniana.* These findings indicate that host cells retain only a single algal species, with a preference for native species, suggesting host compatibility. The positioning style of algae below the host *Paramecium* cortex differed among various *Chlorella* spp., potentially due to variations in the formation of the PV membrane.

As shown in Section 3, it was elucidated that the digestive enzyme resistance of the algae was dependent on the photosynthetic activity of the isolated *C. variabilis* 1N prior to their incorporation with algae-free *P. bursaria* [53]. Moreover, *C. variabilis* NC64A in the logarithmic phase of growth exhibited low infectivity to algae-free *P. bursaria* cells, whereas those in the early stationary phase displayed high infectivity [64]. Consequently, the conditions under which endosymbiosis occurs between *Chlorella* sp. and algae-free *P. bursaria* are progressively being elucidated. These results provide insights into symbiotic interactions and may aid in controlling microbial infections and creating beneficial microorganisms in combination with *P. bursaria*. Additionally, this understanding may be applied to elucidate the processes of bacterial infection in *P. bursaria* and to aid in preventing the dissemination of harmful bacteria that utilize *Paramecium* as an environmental host, such as *Legionella pneumophila* and *F. novicida*, which establish intracellular relationships with *Paramecium caudatum* and *P. bursaria*, respectively [70,72].

## 5. Effects of the Symbiotic *Chlorella variabilis* on Cytoplasmic Crystals and Organelles of the Host *Paramecium bursaria*

### 5.1. Algal Effects on Host Cytoplasmic Crystals

**Figure 4:**
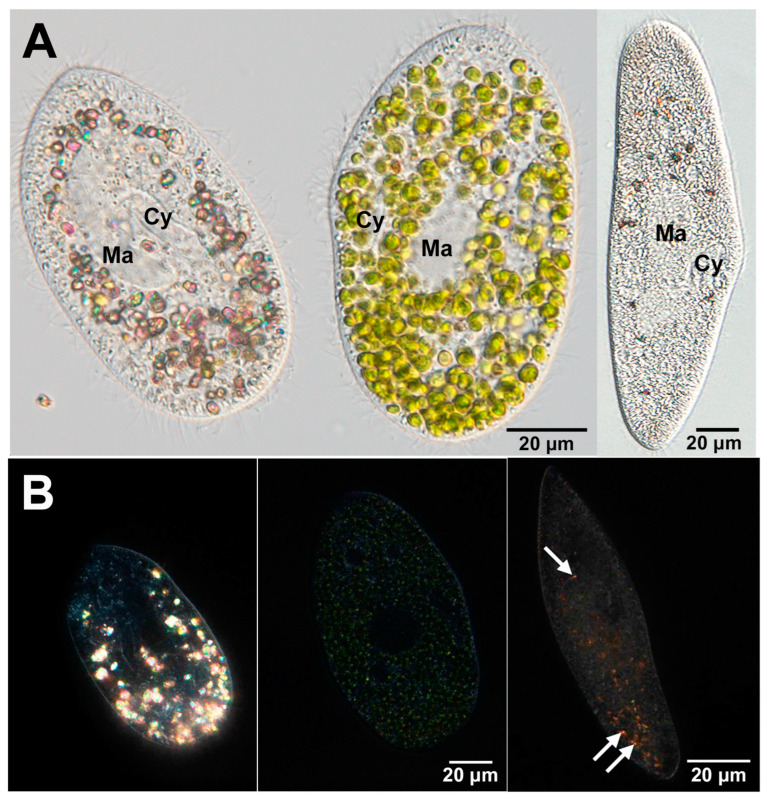
(**A**) DIC micrographs of algae-free *P. bursaria* (left), algae-bearing *P. bursaria* (middle), and *P. multimicronucleatum* (right). Cy, cytopharynx; Ma, macronucleus. (**B**) Highlight the presence of crystals, with increased brightness, to make them more visible. In particular, the posterior end of algae-free *P. bursaria* contained numerous glowing crystals (left), whereas the algae-bearing *P. bursaria* exhibited relatively small crystals (middle). Additionally, a few less bright crystals were present in *P. multimicronucleatum*, as indicated by the white arrow (right). The figure was obtained from [78] and licensed under CC-BY-4.0 International.

Numerous unicellular protists, including ciliates and rhizopods, maintain crystals within the cytoplasm [73], although in both *Tetrahymena pyriformis* and *T. thermophila*, such cytoplasmic crystals observed in other ciliates have not yet been detected (Prof. Dr. Toshiro Sugai, personal communication). Previous studies have elucidated the features of crystals in these protists because they have been easily observed using polarized light microscopy for more than a century [73,74,75]. Transmission electron microscopy (TEM) observations have revealed that cytoplasmic crystals of *Paramecium* sp. are encased in a membrane [76]. However, the origin of the membrane surrounding the crystals remains unknown. Crystals exhibit variations in morphology, and the membrane consistently envelops them. A recent investigation has yielded significant advancements in the study of crystals. Using Raman microscopy, Pilátová et al. [77] identified cellular crystalline inclusions predominantly composed of purines in 77% of more than 200 species across all the major eukaryotic supergroups. Anhydrous guanine crystals have also been observed in *Paramecium* sp. Figure 4A presents the DIC images of symbiotic algae-free and algae-bearing *P. bursaria* (Figure 4A, left and middle) and *P. multimicronucleatum* (Figure 4A, right). *P. multimicronucleatum* is the larger species among the genus *Paramecium*. In a previous study, the solubility of cytoplasmic crystals of *P. multimicronucleatum* was analyzed [73], which enabled a comparative analysis of the prevalence of crystals in *P. bursaria* and *P. multimicronucleatum*. Several green symbiotic *Chlorella* sp. were observed in the algae-bearing *P. bursaria* cells (Figure 4B, middle). In contrast, algae-free *P. bursaria* cells had many polarized orange granules identified as crystals (Figure 4A, left), whereas the algae-bearing cells had almost no crystals (Figure 4A, middle). In algae-free cells, the crystals were mostly localized in the posterior part of the cell (Figure 4B, left). Furthermore, the majority of crystals move via cytoplasmic streaming [69]. *P. multimicronucleatum* (Figure 4B, right) contained only a small number of crystals.

Kodama and Endoh [69] administered various species of *Chlorella* to algae-free *P. bursaria* and examined alterations in the quantity of the crystals within the cells. A significantly reduced abundance of crystals was observed, which were almost entirely absent when the original symbiotic *Chlorella* sp. was used to infect algae-free *P. bursaria*. Conversely, free-living *Chlorella* sp. induced a less pronounced decrease in crystal quantity [69]. This observation suggests the presence of crystal precursors (i.e., guanosine metabolites), which may be a prerequisite for the establishment of suitable endosymbiosis. It could also be that in algae-bearing *P. bursaria*, suitable symbiotic *Chlorella* sp. might alter the metabolism of *P. bursaria*, facilitating the elimination of these guanine crystals.

Kodama et al. [78] examined features of the cytoplasmic crystals of *P. bursaria* in the presence or absence of symbiotic algae and revealed that the length and amount of the crystals depended on the number of symbiotic algae present. Furthermore, symbiotic algae decreased crystal retention in the host cytoplasm. It has been suggested that these crystals may be either excreted from the host cells during algal reinfection or utilized for the maintenance of symbiotic algae. As for the nature of the crystals, it was found that the crystals were soluble in strong bases and acids and were suitable for long-term storage at −20 °C. The significance of this study lies in its providing new perspectives on the role of crystals for *P. bursaria* and how endosymbiotic algae influence their presence. Raman microscopy was utilized to identify cellular crystalline inclusions primarily composed of purines in over 200 species spanning all major eukaryotic supergroups, and it was revealed that approximately 77% of the species exhibited these inclusions [77]. This suggests the possible functions of these crystals in the establishment or maintenance of algal symbioses, such as serving as a storage site for purines and organic nitrogen or detaining nitrogenous waste.

### 5.2. Algal Effects on Host Trichocysts and Mitochondria

**Figure 5:**
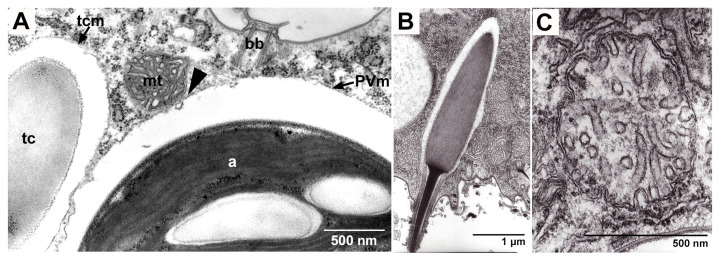
(**A**) Transmission electron micrographs of symbiotic alga below the host cell cortex. The PV membrane (PVm) enclosing the symbiotic alga (a) and the host mitochondria (mt) are in close contact (arrowhead), and the trichocyst membrane (tcm) enclosing the trichocyst (tc) is also present near the PV membrane. The upper parts of the transmission electron micrographs show the cell surface of *P. bursaria*. bb shows the basal body of the host cilia. (**B**,**C**) Transmission electron micrographs of the host trichocyst (**B**) and mitochondrion (**C**). Kodama, unpublished micrographs.

**Figure 6:**
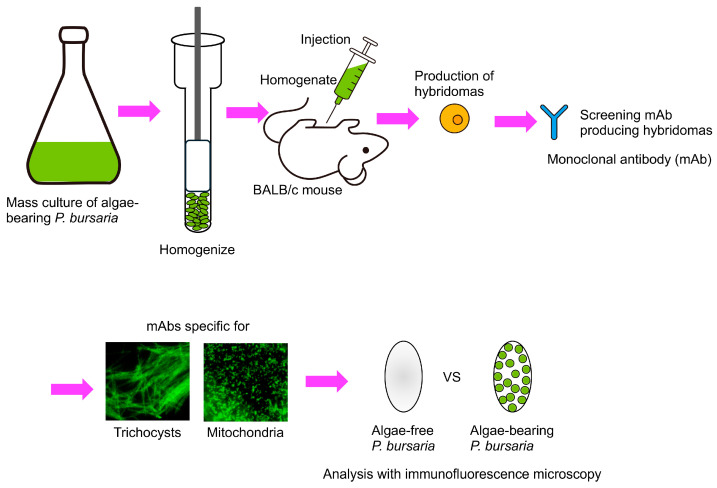
Production of mAbs and experimental flowchart. Mass cultures of algae-bearing *P. bursaria* in the early stationary phase of growth were harvested, and the sedimented cells were homogenized. The homogenate was injected intraperitoneally into 8-week-old BALB/c mice. For hybridoma cell cloning, culture supernatants were screened using indirect IF microscopy [82], and mAbs specific for trichocysts or mitochondria of *P. bursaria* have been developed. Differences in the number and arrangement of host trichocysts and mitochondria before and after algal symbiosis were examined by indirect IF microscopy. This figure was prepared with reference to [61,81].

The PV membrane surrounding the symbiotic *Chlorella* sp. of *P. bursaria* and the host trichocysts and host mitochondria are in close proximity, right below the host cell surface (Figure 5) [79]. The presence of *P. bursaria* mitochondria in close proximity to the PV membrane has also been reported by Song et al. [80]. To investigate the relationship between the symbiotic algae *P. bursaria* and host trichocysts and mitochondria, monoclonal antibodies that specifically recognize the trichocysts or mitochondria of *P. bursaria* have been developed [61,81]. Indirect immunofluorescence (IF) microscopy using these antibodies was conducted to compare the differences in the number of host trichocysts and host mitochondria in the presence or absence of the symbiotic *Chlorella* sp. The experimental flowchart is shown in Figure 6. Many types of mAbs (e.g., those against the DV membrane, symbiotic algal cell wall, macronuclear membrane, micronuclear membrane, cytopharynx, and cytoproct) have been obtained, in addition to those against mitochondria and trichocysts, using the method shown in Figure 6 (Fujishima, unpublished data).

#### 5.2.1. Effects on Host Trichocysts

Thousands of trichocysts are embedded under the *Paramecium* cell surface as defensive organelles against predators [83]. Previous studies have shown that algal symbionts push host trichocysts out of the way and attach themselves to the host cell surface in a location that lacks both trichocysts and acid phosphatase (AcPase) activity, as detected by Gomori’s staining method [11,58]. This indicates that primary lysosomes were not present in this area. These observations suggested that the PV membrane might not have the ability to protect itself from lysosomal fusion, as traditionally said, but could avoid it by localizing to the lysosome-free region of the cell. To confirm this possibility, *P. bursaria* cells were treated with lysozyme to remove trichocysts without seriously damaging the *Paramecium,* and the relationship between the PV membrane, trichocysts, and AcPase activity-negative areas was examined [57]. Our results showed that the PV membrane could protect itself from lysosomal fusion and that trichocysts were not required for this protection. This study is significant because it offers valuable insights into the defensive mechanisms employed by endosymbiotic *Chlorella* sp. to protect themselves from host lysosomal digestion during infection. The mechanism utilized by the PV membrane to prevent algae from undergoing host lysosomal fusion remains unclear and constitutes a significant issue that requires resolution.

Furthermore, Kodama [60] investigated the relationship between *Chlorella* sp. adhesion sites and trichocysts during algal removal and reinfection. During symbiotic algal removal, algae in the anterior cortex were easier to remove than those in the posterior, ventral, and dorsal cortices. In contrast, during algal reinfection, algae tended to localize more readily below the ventral or dorsal cortex than at the other cortices, although there was no localization when trichocysts were completely removed from the host *P. bursaria*. Furthermore, trichocyst discharge experiments revealed that trichocysts in the anterior cortex were difficult to remove. Among the 14 strains of algae-bearing *P. bursaria*, some paramecia lacked symbiotic algae at the anterior cortex, demonstrating the difficulty of localizing algae at this site and, conversely, the ease of removing them from the area. This study sheds light on the mechanisms of algal compartmentalization and localization in endosymbiotic relationships.

**Figure 7:**
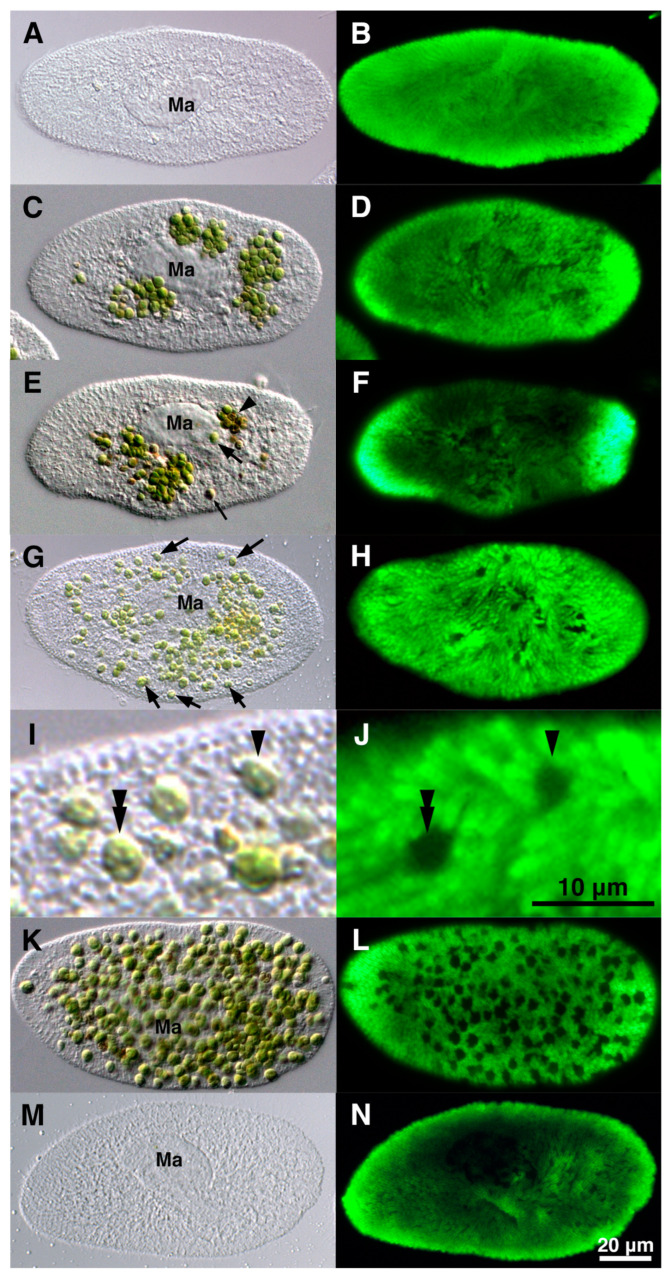
Photomicrographs of algae-free *P. bursaria* during algal reinfection and changes in the number and arrangement of host trichocysts. The images (**A**,**C**,**E**,**G**,**I**,**K**,**M**) are shown as DIC images, and the images (**B**,**D**,**F**,**H**,**J**,**L**,**N**) as IF images. (**I**,**J**) are enlarged images of upper parts of (**G**,**H**). Green fluorescence (that is, IF) indicates the presence of host trichocysts. IF was observed throughout the cells before mixing with the algae (**B**). Thirty minutes after mixing, one of the algae exhibited temporary resistance to host lysosomal enzymes and remained intact in the DV ((**E**), arrowhead). Indirect IF microscopy revealed no alterations in the trichocysts (**F**). Three hours post-mixing, green algae that had escaped from the host DVs were observed to localize below the host *Paramecium* cortex ((**G**); arrows). Trichocysts reorganized into a ring-like formation surrounding the symbiotic algae (arrowhead and double arrowhead in (**J**)). Twenty-four hours after mixing, algae proliferated through cell division, establishing endosymbiosis (**K**). Ring-shaped trichocysts increased concomitantly with algae below the host *Paramecium* cortex (**L**). Forty-eight hours after mixing, some algae-free *P. bursaria* cells did not establish endosymbiosis with the isolated symbiotic algae (**M**), and the IF pattern of the trichocysts resembled that observed under pre-mixing conditions (**N**). Ma, macronucleus. The figure was taken from [61] with permission.

Changes in trichocysts during algal reinfection were investigated using indirect IF microscopy [61]. Prior to mixing with isolated symbiotic algae (Figure 7A), the *P. bursaria* cells displayed numerous trichocysts below the *Paramecium* cortex (Figure 7B). Upon mixing with the isolated symbiotic algae, some algae were enclosed within DV-I via the host cytopharynx (Figure 7C). Indirect IF microscopy revealed no change in the number of trichocysts (Figure 7D). Approximately 0.5 h post-mixing, some algae developed temporary resistance to the host lysosomal enzyme within the DV, which also contained the digested algae (Figure 7E, arrowhead). A single green and digested brown *Chlorella* sp. emerged from the DV membrane (Figure 7E, large and small arrows). Indirect IF microscopy revealed no change in the number of trichocysts (Figure 7F). After escaping DV into the host cytoplasm via membrane budding, the algae were localized immediately below the host *Paramecium* cortex. The percentage of cells with a single green alga below the host *Paramecium* cortex increased over time during reinfection (Figure 7G, arrows). Trichocysts formed rings around each alga below the host *Paramecium* cortex (Figure 7J). Approximately 24 h after mixing, the number of algal cells below the host *Paramecium* cortex further increased (Figure 7K), and the ring-like arrangement of trichocysts increased with an increase in the number of green algae in the host cytoplasm (Figure 7L). Algae-free *P. bursaria* cells, which failed to establish endosymbiosis with the isolated symbiotic algae (Figure 7M), exhibited no change in the trichocyst arrangement (Figure 7N). To reduce the number of host trichocysts and changes in their arrangement, the trichocysts below the host *Paramecium* cortex were locally digested by the host through the fusion of lysosomes to preserve the algal attachment sites during the initial stages of algal reinfection. This study used indirect IF microscopy and transmission electron microscopy to demonstrate that symbiotic algae competed with pre-existing trichocysts for attachment sites and possessed the ability to secure algal attachment sites below the *Paramecium* cortex. It offers valuable insights into the interplay between the host *P. bursaria* and the symbiotic *Chlorella* sp. and how they collaborate to establish a lasting symbiosis. Moreover, this study emphasizes the role of lysosomal fusion in the host digestive system to facilitate the attachment of symbiotic algal sites below the host *Paramecium* cortex.

To investigate the correlation between the number of trichocysts and symbiotic algae, algae-bearing or algae-free *P. bursaria* were subjected to starvation for several days, and alterations in the number of symbiotic algae and trichocysts were evaluated [84]. Indirect IF microscopy using an anti-trichocyst mAb revealed that under starvation and dark conditions, the IF of trichocysts in algae-free *P. bursaria* diminished at a significantly higher rate than that in algae-bearing *P. bursaria*. This suggests that the nutrients obtained from symbiotic algal digestion may promote the synthesis or retention of trichocysts. This finding suggests that the host *P. bursaria* gains new benefits from harboring symbiotic algae, which could have implications for understanding the ecology and evolution of symbiotic relationships in nature.

Recently, Morita and Kodama [85] conducted a comparative analysis of the regeneration capacity of trichocysts in algae-free and algae-bearing *P. bursaria* [85]. *Additionally, trichocyst protein abundance was measured in P. bursaria* specimens artificially infected with *Chlorella* sp. to algae-free *P. bursaria*. Following the complete removal of trichocysts from *P. bursaria* cells using lysozyme and subsequent examination after 24 h, the percentage of regenerating trichocysts in the entire cell was significantly higher in algae-free cells than in algae-bearing cells. There are three potential explanations for the observed phenomenon. First, as suggested by Ehret and McArdle [86], the absence of trichocysts in algae-free *P. bursaria* cells may be attributed to their capacity to regenerate these structures at a faster rate to maintain the strength and integrity of the *Paramecium* cortex. The presence of symbiotic algae may contribute to the reinforcement of the surface structure of *P. bursaria*, potentially substituting for trichocysts. Second, the abundance of symbiotic *Chlorella* sp. adhering to the cell cortex in algae-bearing *P. bursaria* may limit the space available for trichocysts to adhere. This notion is supported by the findings of [57], which demonstrated that the removal of trichocysts increased the surface area available for the symbiotic *Chlorella* sp. to attach to the *Paramecium* cortex, leading to an increase in the number of symbiotic algae in the host. Consequently, the resynthesis of trichocysts in algae-bearing *P. bursaria* occurs at a slower rate than that in algae-free cells because of the limited space available for trichocyst reattachment. Third, Berger [87] postulated that *Didinium nasutum*, a predator of *Paramecium* spp., demonstrated a preference for attacking and ingesting *P. bursaria* devoid of algae, as opposed to those containing algae. The symbiotic algae present within *P. bursaria* are hypothesized to secrete unpalatable metabolites that deter *D. nasutum*, thereby conferring a protective function to the host [87]. Given that the primary function of trichocysts is to defend against predators [83], algae-free *P. bursaria* may require the rapid regeneration of trichocysts to mitigate predation. However, additional empirical studies are needed to validate this hypothesis. Morita and Kodama [85] developed a simplified method for isolating high-purity trichocysts to quantify trichocyst protein amounts. This study revealed a significant difference in trichocyst protein abundance in *P. bursaria* before and one week after introduction to *Chlorella* sp. (i.e., following the establishment of symbiosis with the algae).

#### 5.2.2. Effects on Host Mitochondria

As shown in Section 5.2.1., trichocysts were not considered necessary for the attachment of the symbiotic *Chlorella* sp. immediately below the cell surface layer. Based on TEM, it has been proposed that host mitochondria near the *Paramecium* cortex might play a role in the localization of PVs within this region [79], and mAbs have been developed for *P. bursaria* mitochondria to test this hypothesis and clarify the algal localization mechanism. Mitochondria are well known to play crucial roles in energy production, cell-autonomous immunity, and apoptosis [88]. Investigating the relationship between symbiotic or parasitic organisms and host mitochondria is important because it provides insights into the complex interactions that underpin host–pathogen dynamics.

**Figure 8:**
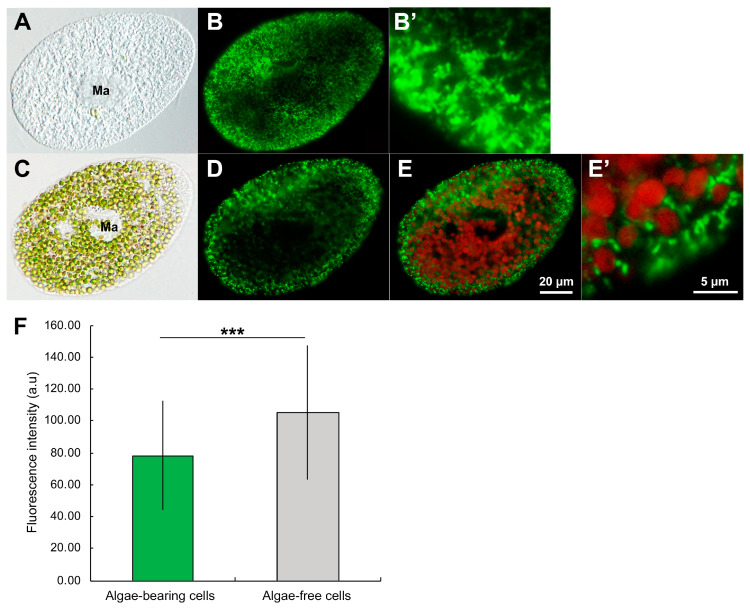
DIC and indirect IF photomicrographs of symbiotic algae-free and algae-bearing *P. bursaria* labeled with a mAb specific for *Paramecium* mitochondria. (**A**,**B**,**B’**): algae-free *P. bursaria*. (**C**), (**D**,**E**,**E’**): algae-bearing *P. bursaria*. (**A**,**C**): DIC photomicrographs. (**B**–**E**,**E’**): indirect IF micrographs. Small granules with green fluorescence indicate the host mitochondria. Large particles with red fluorescence show autofluorescence of chlorophyll in algal chloroplasts. (**B’**): an enlarged view of the host cortex in (**B**). (**E**): a merge of (**D**) and the autofluorescence of chlorophyll. (**E’**): enlarged view of (**E**), in which several symbiotic algae are surrounded by host mitochondria. It should be noted that the mitochondria near the cell cortex in algae-free cells (**B’**) were more abundant than those in algae-bearing cells (**E’**). Ma, macronucleus. Scale bars for (**A**–**E**) represent 20 μm, and those for (**B’**,**E’**) represent 5 μm. (**F**): IF intensity of mitochondria in algae-free and algae-bearing *P. bursaria*. In algae-free *P. bursaria* (gray bar graph), the IF intensity was higher than that in algae-bearing *P. bursaria* cells (green bar graph). Error bars represent standard deviation (SD). Asterisks denote significant differences (two-sided Fisher’s exact test; *** *p* < 0.001). These photomicrographs and a graph were obtained from [81] and licensed under CC-BY-4.0 International.

A mAb that specifically targets *Paramecium* mitochondria has been developed [81]. Indirect IF with a mAb displayed many mitochondria throughout *P. bursaria* cells, with a particularly high concentration near the cell cortex, as shown in Figure 8B,D. In contrast, the mitochondrial fluorescence was low in the central regions containing symbiotic algae when this technique was applied to algae-bearing cells (Figure 8D). This demonstrates a greater abundance of mitochondria in algae-free cells than in algae-bearing cells. The combined image of chlorophyll red autofluorescence within the symbiotic algal chloroplasts and indirect IF revealed the exact positioning of the host mitochondria encircling the symbiotic algae, as shown in Figure 8E,E’. This study revealed the substantial influence of symbiotic algae on the quantity of mitochondria in the host. The IF intensity of algae-free *P. bursaria* (Figure 8F, gray bar graph) exhibited a higher magnitude than that of algae-bearing cells (Figure 8F, green bar graph) [81]. This result was supported by the fact that the overall cell protein content in algae-free *P. bursaria* cells was roughly 1.8 times higher than that in algae-bearing cells; in particular, mitochondrial protein content was notably elevated in algae-free cells compared with algae-bearing cells [81]. Thus, symbiotic algae have a substantial effect on the reduction in host mitochondrial numbers, suggesting that host cells modify mitochondrial density through an unknown mechanism to accommodate algae.

Commercial fluorescent dyes, such as MitoTracker Green (Molecular Probes, Eugene, OR, USA) [81] and MitoBright LT Green (Dojindo Laboratories, Kumamoto, Japan), can also be utilized to visualize mitochondria in *P. bursaria* (Kodama, unpublished data). However, the specificity of mitochondrial labeling was hindered because DVs that ingested these dyes were also formed. This is due to the uptake of anything other than food by the *Paramecium* through the cytopharynx, even the fluorescent dyes. The same phenomenon did not occur when mAbs were used, indicating the utility of mAbs for mitochondrial visualization, such as ciliates with highly active phagocytic activity.

The presence of host mitochondria in close proximity to symbionts or parasites may be a common feature of symbiotic or parasitic relationships [89], as shown in *Mayorella visiris* [90,91], *Toxoplasma gondii* [92,93], *Plasmodium sporozoites* [94], and *Encephalitozoon* microsporidia [95]. Candidatus *Midichloria mitochondrii*, a symbiont inherited through maternal lines, has the ability to inhabit the mitochondria of oocytes in *Ixodes ricinus*, one of Europe’s most common tick species [96]. These microorganisms are found not only in the cytoplasm but also within the mitochondrial intermembrane space. Their presence in this location appears to result in condensation of the mitochondrial matrix [97]. In ciliates, the presence of bacterial-like inclusions within the mitochondria is extremely rare and has been identified in only three species: *Spirostomum minus* [98]; *Halteria geleiana* [99]; and *Urotricha ovata* [100]. The importance and role of host mitochondria in the symbiotic *Chlorella* sp. of *P. bursaria* remains largely unknown. Research on the mutualistic association between cnidarians and their *Symbiodinium* spp. dinoflagellate partners revealed that excessive heat stress caused the breakdown of cnidarian host mitochondria independent of symbiont cell deterioration [14]. This process is characterized by a notable decrease in the expression of host cytochrome c and ATP synthase. Disruption of the host mitochondrial function, cellular equilibrium, and subsequent cell death may trigger coral bleaching. These findings offer crucial insight into the mechanisms responsible for the collapse of this symbiotic relationship [101]. Examining the impact of symbionts on the quantity and function of organelles in host protists is crucial for understanding the complex symbiotic relationships and their effects on host cell physiology. Symbionts can modify host gene expression, contribute to metabolic activities, and shape host immune responses [102], potentially influencing the function and number of host organelles through various pathways. For example, research on the social amoeba *Dictyostelium discoideum* has shown that the presence of symbiotic bacteria was linked to genomic adaptations that might affect host-symbiont interactions, possibly including changes in organelle function [103].

### 5.3. Algal Effects on Host Trichosysts, Mitochondria, Cytoplasmic Crystals, Total Protein Amount, Heat Tolerance, Photoaccumulation, and Circadian Rhythms

Table 1 summarizes the changes in host *P. bursaira* before and after algal endosymbiosis. Notably, the abundance of trichocysts [57,61,81], mitochondria [81], cytoplasmic crystals [78], and total cell protein [81] was decreased in algae-bearing *P. bursaria*. The trichocyst regeneration speed decreased in algae-bearing *P. bursaria* [85]. Iwatsuki et al. [104] and Miwa [24] obtained almost the same result that symbiotic *Chlorella* enhanced the tolerance to high temperature (41–42 °C) in *P. bursaria*, and algae-bearing cells showed a higher survival rate than algae-free cells in constant light condition. Iwatsuki and Naitoh [105] reported behavioral responses to light in *P. bursaria* and identified three types of photoreceptor systems. Matsuoka and Nakaoka [106] showed that photoaccumulation, a phenomenon in which cells gather in the light-hit area, appeared at peaks at wavelengths of approximately 420 nm (blue–purple) and 560 nm (yellow–green) in both algae-bearing and algae-free cells at a constant light intensity of 0.5 mW/cm^2^. Furthermore, they showed that in both algae-bearing and algae-free cells, 420 nm light increased photoaccumulation with increasing light intensity at about 0.2 mW/cm^2^ or less, and 560 nm light increased photoaccumulation with increasing light intensity at about 2 mW/cm^2^ or less. Under these two light wavelength conditions, further increasing the light intensity decreased light accumulation, and the cells accumulated right outside the light spot, avoiding the inside of the spot. The fact that algae-free cells are attracted to light of wavelengths (400 to 700 nm) and intensities suitable for photosynthesis indicates that *P. bursaria* has the ability necessary to maintain endosymbiosis with algae. Their results also indicate that at light intensities of less than 0.1 mW/cm^2^ at 420 nm, the photoaccumulation of the algae-free cells was higher than that of the algae-bearing cells, and at more than 2 mW/cm^2^ at 560 nm, the photoaccumulation of the algae-free cells was higher than that of the algae-bearing cells. Thus, endosymbiotic algae can affect the host photoaccumulation at specific light intensities and wavelengths. However, they used different strains of *P. bursaria* for algae-bearing and algae-free cells; some differences in genetic backgrounds or degrees of aging between these two strains may have affected the differences in their photoaccumulations. Thus, algae-free *P. bursaria* also has a photoaccumulation ability, and the wavelengths of light required for photoaccumulation coincide with those required for photosynthesis [106,107]. Johnson et al. [108] examined the photoaccumulation ability of *P. tetraurelia*, *P. caudatum*, and *P. multimicronucleatum*, which cannot establish endosymbiosis with algae, and found that none of them had the photoaccumulation ability. Similarly, the ciliate *Blepharisma japonicum*, which also cannot bear algae in its cytoplasm, showed no photoaccumulation. These results strongly suggest that photoaccumulation is a prerequisite for the indispensable ability of host cells to establish endosymbiosis with algae.

**Figure 9:**
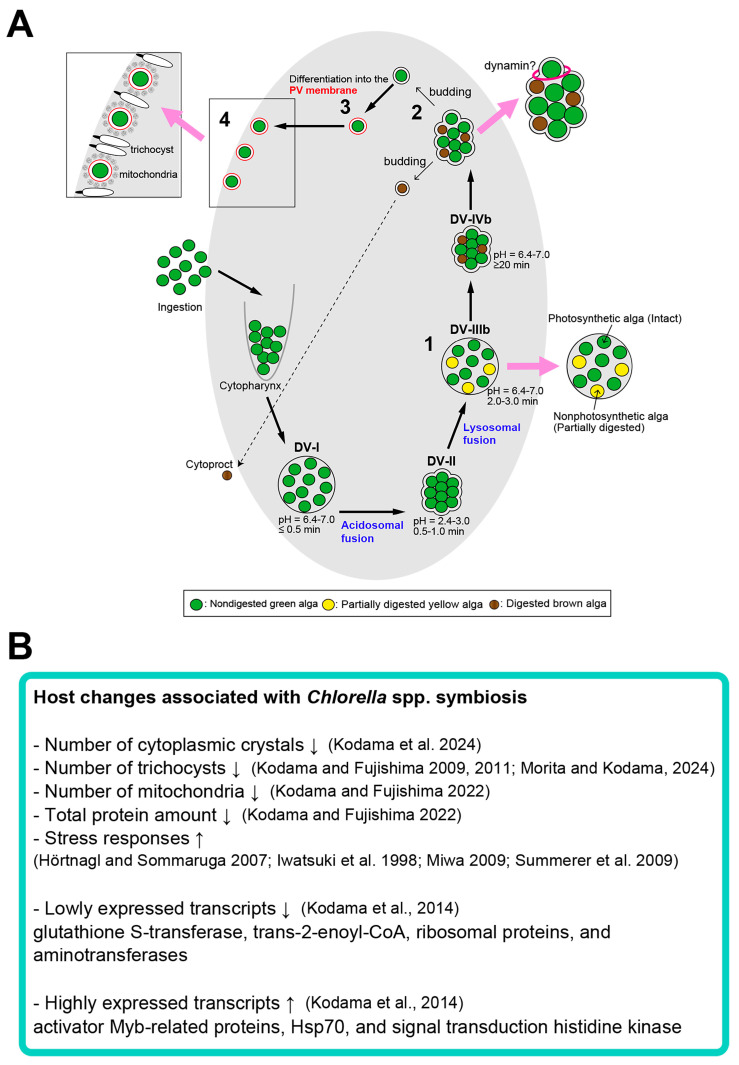
(**A**) Schematic representation of the algal reinfection. Four important cytological events (labeled 1–4) are required to establish endosymbiosis [51]. 1. Approximately 3 min after mixing algae-free *P. bursaria* cells with isolated symbiotic algae, some algae in the DVs acquire resistance to host lysosomal enzymes. 2. Approximately 30 min after mixing, algae escape from the DVs by budding the DV membrane. This budding is completely inhibited by the dynamin inhibitor Dynasore. 3. Approximately 45 min after mixing, the DV membrane enclosing a single undigested green alga differentiates into the PV membrane (red), which provides protection from host lysosomal fusion. 4. The alga localizes beneath the host cell cortex by the adhesion of the PV membrane to the host mitochondrial outer membrane and pushing aside host trichocysts. Approximately 24 h after mixing, the alga beneath the host cell cortex begins cell division and establishes endosymbiosis (updated from [112] with permission). (**B**) Host changes associated with algal symbiosis. References numbers are as follows. Kodama et al. 2024 [78]; Kodama and Fujishima 2009 [57]; Kodama and Fujishima 2011 [61]; Morita and Kodama 2024 [85]; Kodama and Fujishima 2022 [81]; Hörtnagl and Sommaruga 2007 [23]; Iwatsuki et al. 1998 [104]; Miwa 2009 [24]; Summerer et al. 2009 [25]; Kodama et al., 2014 [42].

Miwa [24], Miwa et al. [109], and Tanaka and Miwa [110] showed that the symbiotic *Chlorella* of the *P. bursaria*, by using the photosynthetic product maltose, played a leading role in host circadian rhythms of photoaccumulation and mating reactivity in the following three respects: (1) prolongation of period length; (2) induction of phase shift; and (3) expression of mating reactivity rhythms in arrhythmic mutants. Furthermore, Miwa [24] suggested that although mitochondria in host *P. bursaria* also showed a rhythm in morphological changes and division, the clock of *Chlorella* was superior to that of the host *Paramecium* and host mitochondrial clocks. Microbial symbioses can have both positive and negative effects on host organisms. The study of symbiont effects on host behavior, physiology, and phenotype is important because it also provides insights into the complex interactions that influence host fitness and ecosystem processes [111]. Figure 9 presents a summary of this review.

## 6. Conclusions

In this review, it was concluded that the symbiotic *Chlorella* sp. decreased the number of host cytoplasmic crystals, trichocysts, and mitochondria. Furthermore, this review summarizes the effects of symbiotic algae on the total protein amount, stress responses, photoaccumulation, and circadian rhythms of the host *P. bursaria*. Future studies should explore the mechanisms underlying the observed phenomena in the symbiotic relationship between *P. bursaria* and *Chlorella* spp. Based on previous experiments on the photoaccumulation capacity of the host cell and successful endosymbiosis with green algae, a hypothesis is that the possession of photoaccumulation capacity by the host cell is a prerequisite for achieving endosymbiosis with algae. Elucidating when and how eukaryotic cells such as *P. bursaria*, which can establish endosymbiosis with algae, acquire this ability is necessary not only for elucidating the mechanisms of cellular evolution but also for creating useful cells with new photosynthetic capacities. *Paramecium* species have two kinds of nuclei, a germinal micronucleus and a somatic macronucleus, in a cell, and the cell can grow even after removing the micronucleus [113,114,115]. However, the influence of the presence or absence of micronuclei on host phenotypes induced by symbiotic algae remains unclear. In summary, future research should focus on the molecular and cellular mechanisms that enable and maintain the symbiotic relationship between *P. bursaria* and *Chlorella* spp., the influence of symbiosis on host cell structure and function, and the environmental factors that affect the symbiotic system. These studies may provide a deeper understanding of the symbiotic interactions and their evolutionary implications.

## Figures and Tables

**Table 1:** Phenotypic changes in the host *P. bursaira* before and after algal endosymbiosis.

Phenomena	Algae-Free *P. bursaria*	Algae-Bearing *P. bursaria*	References
Abundance of trichocysts	Increase	Decrease	[57,61,81]
Trichocyst regeneration speed	Increase	Decrease	[85]
Abundance of mitochondria	Increase	Decrease	[81]
Abundance of cytoplasmic crystals	Increase	Decrease	[78]
Abundance of total cell protein	Increase	Decrease	[81]
Heat tolerance	Decrease	Increase	[24,104]
Photoaccumulation	Almost no change	Almost no change	[105,106]
Circadian rhythms on photoaccumulation and mating reactivity	Depend on the host clock	Depend on the *Chlorella* clock under constant light conditions	[24,109,110]

## Data Availability

Data sharing is not applicable to this article, as no new data were created or analyzed in this study.
